# Intramedullary Nails vs. Locking Plates for Displaced Proximal Humerus Fractures in Patients over 60: A Comparative Clinical Study

**DOI:** 10.3390/jcm14134563

**Published:** 2025-06-27

**Authors:** Marco Simone Vaccalluzzo, Marco Sapienza, Sergio Valenti, Benedetta Di Tomasi, Ludovico Lucenti, Vito Pavone, Gianluca Testa

**Affiliations:** 1 Department of General Surgery and Medical Surgical Specialties, Section of Orthopaedics, A.O.U. Policlinico Rodolico-San Marco, University of Catania, 95123 Catania, Italy; marcovaccalluzzo@hotmail.it (M.S.V.); marcosapienza09@yahoo.it (M.S.); sergio.valenti15@gmail.com (S.V.); benedettadtm@gmail.com (B.D.T.); vitopavone@hotmail.com (V.P.); 2 Department of Precision Medicine in Medical, Surgical and Critical Care (Me.Pre.C.C.), University of Palermo, 90133 Palermo, Italy; ludovico.lucenti@gmail.com

**Keywords:** humeral fractures, intramedullary fracture fixation, bone plates, shoulder fractures, recovery of function

## Abstract

**Background:** Displaced proximal humerus fractures (PHFs) in adults represent a common orthopedic challenge, especially in elderly patients with compromised bone quality. Intramedullary nails (IM) and locking plates (LP) are the most widely used fixation techniques, though the literature remains inconclusive regarding their comparative efficacy. **Methods:** This retrospective cohort study included 187 patients (mean age: 65.4 years) treated surgically for Neer ≥ 2-part proximal humerus fractures at a single tertiary referral center between 2018 and 2023. Patients underwent either IM nailing or LP fixation. Baseline characteristics included age, sex, smoking status, ASA score, trauma mechanism, and bone quality (assessed by the Deltoid Tuberosity Index). Functional outcomes (DASH and Constant–Murley scores), range of motion (ROM), radiographic healing, and complications were evaluated at 1, 3, 6, and 12 months postoperatively. Results were stratified by fracture type (two-, three-, and four-part fractures) and treatment group. **Results:** At 12 months, no statistically significant differences were found between groups in terms of DASH (*p* = 0.484) or Constant–Murley scores (*p* = 0.057). ROM recovery was comparable across all time points. Stratified analysis showed similar outcomes across fracture types. Age, smoking, and bone quality did not significantly influence clinical results. The overall complication rate was 11.8%, with no significant difference between groups. Avascular necrosis and hardware-related issues occurred predominantly in four-part fractures. **Conclusions:** Both intramedullary nailing and locking plate fixation provided comparable short-term outcomes for displaced PHFs. Functional recovery appeared more dependent on fracture complexity than on the choice of implant. Surgical technique should therefore be selected based on fracture morphology, patient characteristics, and surgeon experience.

## 1. Introduction

Proximal humerus fractures (PHFs) are among the most common fractures encountered in clinical practice, with an incidence that is expected to rise significantly due to the increase in the aging population. Representing approximately 4–10% of all fractures [1,2], PHFs are the second most prevalent upper extremity fractures and the third most frequent fractures overall in the elderly. Epidemiological data indicate an incidence of 31 to 250 cases per 100,000 adults, with a steady increase correlating with aging demographics. According to Roux et al. [3], the critical age for this type of fracture is 70 years, and the dominant arm is affected in 48% of cases.

Proximal humerus fractures follow a bimodal distribution resulting from low-energy trauma (e.g., falls) in elderly individuals with osteoporosis and high-energy trauma (e.g., road accidents or sports injuries) in younger patients [3,4]. Studies report that in men, 55% of fractures result from simple falls, whereas 45% are due to high-energy trauma, while in women, 82% of fractures are attributed to low-energy falls [5]. The mechanisms leading to PHFs involve direct trauma or an axial load transmitted to the humerus, often through the extended elbow, hand, or forearm [6].

Approximately 80–85% of PHFs are non-displaced or minimally displaced and are managed conservatively, while the remaining 15–20% require surgical intervention, particularly when associated with glenohumeral dislocation. In displaced fractures, surgical treatment is recommended based on several key factors, including fracture pattern (number of fragments, displacement degree, risk of osteonecrosis), patient characteristics (age, functional demand, comorbidities, and bone quality), and surgeon expertise in shoulder trauma management [7,8,9,10].

A variety of surgical options are available for PHFs, including percutaneous K-wires, external fixation, minimally invasive plate osteosynthesis (MIPO), open reduction and internal fixation (ORIF) with locking plates or intramedullary nails, hemiarthroplasty, total shoulder arthroplasty, and reverse total shoulder arthroplasty. However, there remains no universally accepted surgical algorithm, as clinical outcomes are influenced by multiple patient- and fracture-specific factors [9,11,12].

Among the most used surgical techniques, locking plates and intramedullary nails have demonstrated good functional outcomes in properly selected patients. Locking plate osteosynthesis, particularly using Non-Contact Bridging (NCB) plates, is often favored for its ability to provide rigid fixation and preserve vascular supply, while intramedullary nail fixation offers a minimally invasive approach with biomechanical advantages [8,9,13,14].

Despite the widespread use of these techniques, comparative clinical evidence remains limited, and no definitive consensus exists regarding the optimal fixation method for displaced PHFs.

The objective of this study is to compare the clinical and functional outcomes of locking plate osteosynthesis (NCB plates) and intramedullary nail fixation in patients with displaced proximal humerus fractures. To achieve this, we analyzed DASH and Constant–Murley scores as measures of functional recovery, along with range of motion (ROM) at 1, 3, 6, and 12 months postoperatively. Given the observed baseline age difference between the treatment groups, a stratified analysis was performed to evaluate whether age significantly influenced clinical outcomes. This approach aimed to reduce potential confounding effects and clarify whether implant choice or patient age was more determinant in functional recovery.

## 2. Materials and Methods

### 2.1. Study Design and Ethical Approval

This retrospective cohort study was conducted at the Department of Orthopaedics, A.O.U. Policlinico Rodolico–San Marco, University of Catania, between 2018 and 2023. All procedures were performed in accordance with the principles outlined in the Declaration of Helsinki and received approval from the Institutional Review Board of the University of Catania (approval number: 160/2020/PO). All patients provided written informed consent prior to participation.

A total of 245 patients with proximal humerus fractures were initially screened. After applying the inclusion and exclusion criteria, 58 patients were excluded for the following reasons: pathological fractures (*n* = 12), open fractures (*n* = 9), prior surgery on the affected shoulder (*n* = 7), severe cognitive impairment (*n* = 10), and loss to follow-up before 12 months (*n* = 20). The final cohort included 187 patients who were assigned to two treatment groups: 96 patients underwent intramedullary nail fixation (IM group) and 91 patients received locking plate fixation (LP group). Data were extracted from electronic medical records and patient charts. A flow diagram illustrating patient selection is presented in the Results section.

### 2.2. Inclusion and Exclusion Criteria

Patients were eligible for inclusion if they were 18 years or older, had a displaced proximal humerus fracture classified as Neer ≥ 2-part, underwent surgical fixation with either an intramedullary nail or a locking plate within 14 days of the trauma, and completed at least 12 months of clinical and radiographic follow-up. Exclusion criteria included pathological or open fractures, head-splitting fractures requiring primary arthroplasty, prior surgery on the affected shoulder, severe cognitive impairment, and failure to complete the follow-up period.

### 2.3. Surgical Technique

All procedures were performed by orthopedic trauma surgeons with expertise in upper limb fixation with patients placed in the beach-chair position under general anesthesia.

In the IM group, patients were treated using the Citieffe^®^ Proximal Humerus Nail (Citieffe, Bologna, Italy), which was inserted through an antegrade, rotator cuff-sparing approach. Proximal and distal locking screws were placed to ensure angular and axial stability following the manufacturer’s recommendations.

In the LP group, fixation was achieved with the Non-Contact Bridging (NCB^®^) Proximal Humerus Plate (Zimmer Biomet, Warsaw, IN, USA) inserted via a standard deltopectoral approach. Anatomical fracture reduction was confirmed under fluoroscopic guidance and stabilized with locking screws.

Postoperative radiographs were obtained for all patients to verify correct implant positioning. The decision to use a nail or plate was based on fracture morphology (Neer classification), bone quality (Deltoid Tuberosity Index), patient characteristics (including age and comorbidities), and the operating surgeon’s preference. Both implants were employed across different fracture types, reflecting the variability in clinical practice.

### 2.4. Postoperative Rehabilitation Protocol

All patients followed a standardized rehabilitation program. From weeks 0 to 3, the shoulder was immobilized with a sling and passive mobilization was initiated, with flexion and abduction limited to 90° and rotation allowed only within pain-free limits. Between weeks 3 and 6, patients began active-assisted range-of-motion exercises. After 6 weeks, they progressed to full active movements and strengthening exercises. By 12 weeks, patients resumed daily activities, with a gradual return to sport activities as tolerated.

### 2.5. Baseline Characteristics and Bone Quality

Demographic and clinical baseline variables recorded included patient age, sex, American Society of Anesthesiologists (ASA) score, smoking status, trauma mechanism (low- or high-energy), affected side and dominance, Deltoid Tuberosity Index (DTI) as a proxy for bone quality, time from injury to surgery (mean: 5.2 ± 1.4 days), and the presence of ongoing osteoporosis treatment at the time of fracture. Fractures were classified according to the Neer classification system by two independent reviewers. Any discrepancies were resolved by consensus. Dual-energy X-ray absorptiometry (DEXA) scans were not routinely performed due to the retrospective nature of the study and limited availability in the acute trauma setting.

### 2.6. Clinical and Functional Assessment

Functional outcomes were assessed at 1, 3, 6, and 12 months postoperatively using the Disabilities of the Arm, Shoulder, and Hand (DASH) score and the Constant–Murley Score (CMS).

Shoulder range of motion (ROM) was assessed using active movement and included forward flexion, abduction, internal rotation, and external rotation. A standard universal plastic goniometer with 360° scale was used for the measurements following the recommendations of the American Academy of Orthopaedic Surgeons. All assessments were performed with the patient in a seated position by two independent orthopedic surgeons trained in shoulder examination.

### 2.7. Radiographic Evaluation and Complications

Radiographs in the anteroposterior and lateral views were obtained at each follow-up. Radiographic outcomes were categorized as follows: union was defined by bridging callus visible on at least three cortices; non-union was defined by lack of healing after six months; malunion was defined by angulation greater than 20° or displacement greater than 5 mm; and avascular necrosis (AVN) was diagnosed radiographically and confirmed with MRI when clinically suspected.

All complications were recorded and classified into the following categories: superficial or deep infections, non-union or malunion, avascular necrosis, hardware-related complications (e.g., screw loosening or nail migration), and need for reintervention (e.g., hardware removal or revision surgery).

### 2.8. Statistical Analysis

All analyses were performed using IBM SPSS Statistics version 30.0 (IBM Corp., Armonk, NY, USA). The normality of continuous variables was tested using the Shapiro–Wilk test. Continuous variables were expressed as mean ± standard deviation (SD) and compared using independent *t*-tests or Mann–Whitney U tests, as appropriate. Categorical variables were compared using chi-square or Fisher’s exact tests. Pearson’s correlation was applied to assess associations between age and functional outcomes. To adjust for potential confounders (age, fracture type, DTI, smoking status), multivariable linear regression models were applied. All statistical tests were two-tailed, and a *p*-value < 0.05 was considered statistically significant. Ninety-five percent confidence intervals (95% CI) were calculated for all primary outcomes.

## 3. Results

### 3.1. Demographic Data

**Figure 1:**
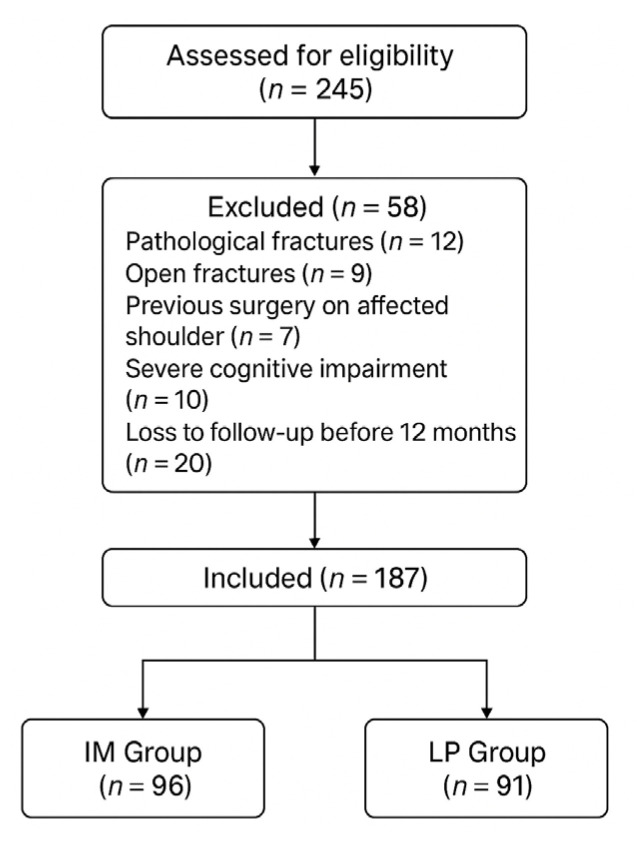
Flow diagram illustrating the patient selection process. A total of 245 patients were initially screened. Following the application of inclusion and exclusion criteria, 58 patients were excluded and 187 were included in the final analysis.

A total of 245 patients were initially assessed. After applying inclusion and exclusion criteria, 58 patients were excluded and 187 were included in the final analysis, as shown in the flow diagram (Figure 1).

The mean age was significantly higher in the IM group compared to the LP group (68.2 ± 7.0 vs. 62.4 ± 9.0 years, *p* < 0.001). The gender distribution was comparable between the groups (IM: 39 males and 57 females; LP: 35 males and 56 females; *p* = 0.879).

Fractures were classified according to the Neer system. In the IM group, 36.5% were two-part fractures, 41.5% three-part, and 22.0% four-part. In the LP group, 27.5% were two-part, 44.0% three-part, and 28.5% four-part fractures. No significant differences were observed between the two groups in fracture pattern distribution.

Bone quality assessment based on the deltoid tuberosity index (DTI) revealed that 33.3% of patients in the IM group and 23.1% in the LP group had a DTI < 1.4, indicating low bone density (*p* = 0.108).

The average time from trauma to surgery was 5.1 ± 1.3 days in the IM group and 5.3 ± 1.4 days in the LP group, with no statistically significant difference (*p* = 0.392).

Other potential confounding variables—including smoking status, comorbidities such as diabetes, body mass index (BMI), and osteoporosis treatment—did not significantly differ between the groups and are reported in Table 1.

### 3.2. Overall Functional Comparison Between Groups

Functional outcomes were assessed at 12 months postoperatively using the Disabilities of the Arm, Shoulder and Hand (DASH) score and the Constant–Murley Score (CMS).

As shown in Table 2, no statistically significant differences were observed between the intramedullary nail (IM) and locking plate (LP) groups across the entire cohort.

The mean DASH scores were 20.6 ± 6.1 in the IM group and 21.2 ± 5.3 in the LP group (*p* = 0.484), while the Constant–Murley Scores were 79.6 ± 8.0 and 77.5 ± 7.6, respectively (*p* = 0.057). The overlap of 95% confidence intervals further confirmed the comparability of the two techniques.

**Figure 2:**
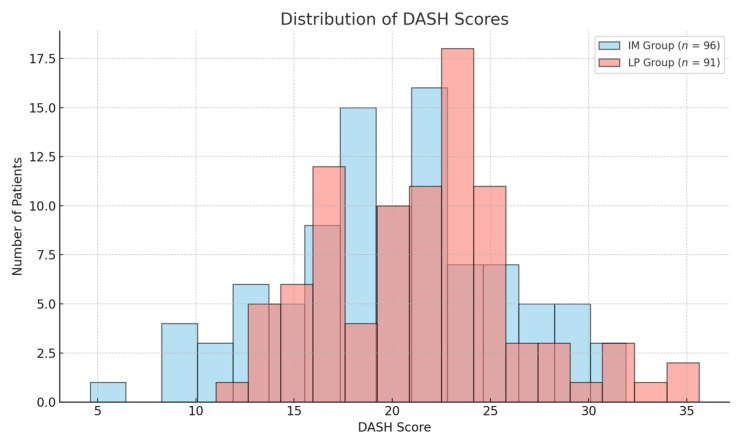
Histogram displaying the distribution of DASH Scores for both IM and LP groups. The distributions are similar, indicating comparable functional outcomes.

**Figure 3:**
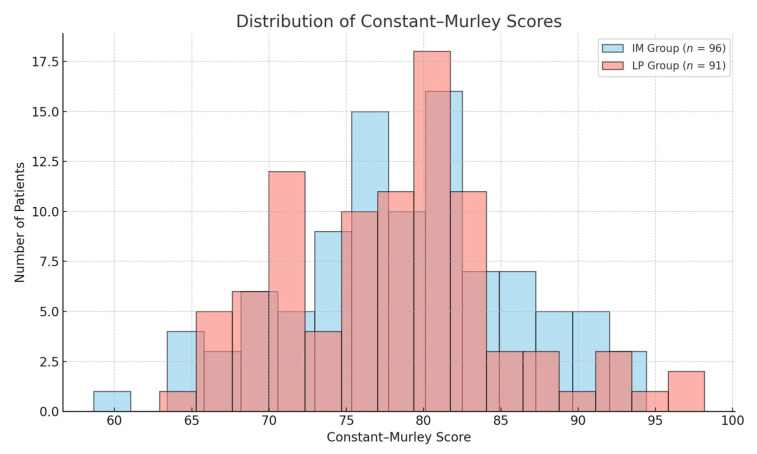
Histogram displaying the distribution of Constant–Murley Scores for both IM and LP groups. No significant differences were observed between the groups.

The distribution of these scores is illustrated in Figure 2 and Figure 3, where the boxplots illustrate the distribution of DASH and Constant–Murley scores across the two groups, confirming the absence of significant differences.

### 3.3. Stratified Analysis by Neer Classification

To investigate the influence of fracture complexity on functional recovery, patients were stratified according to Neer classification into two-part, three-part, and four-part fractures.

As shown in Table 3, both groups demonstrated similar functional scores within each fracture type, with no statistically significant differences. For instance, in two-part fractures, the DASH scores were 19.8 ± 5.2 in the IM group versus 20.3 ± 5.5 in the LP group (*p* = 0.642), and the CMS scores were 82.1 ± 6.2 versus 80.5 ± 6.8 (*p* = 0.318). Results remained comparable in the three-part and four-part subgroups, suggesting consistent outcomes regardless of fracture complexity.

### 3.4. Stratified Analysis by Age Group

To assess the impact of patient age on functional outcomes, a subgroup analysis was conducted comparing patients aged <65 years and ≥65 years. As shown in Table 4, no significant differences were observed between treatment groups in either age category. In patients <65 years, the mean DASH scores were 20.1 ± 5.2 in the IM group and 20.6 ± 5.5 in the LP group (*p* = 0.641), while the CMS scores were 80.8 ± 6.9 and 80.2 ± 7.1, respectively (*p* = 0.578). Among patients aged ≥65 years, the DASH score was 21.9 ± 5.8 (IM) versus 22.0 ± 5.9 (LP) (*p* = 0.934), and the CMS scores were 77.5 ± 8.1 versus 76.2 ± 8.7 (*p* = 0.401).

These findings confirm that both surgical techniques offer similar functional outcomes, independent of patient age.

A correlation analysis was conducted to determine whether age influenced functional recovery:

No significant correlation was found between age and DASH score (r = 0.001, *p* = 0.986).

No significant correlation was found between age and Constant–Murley Score (r = −0.042, *p* = 0.565).

These findings suggest that age does not significantly impact functional recovery in patients treated with either intramedullary nails or locking plates.

**Figure 4:**
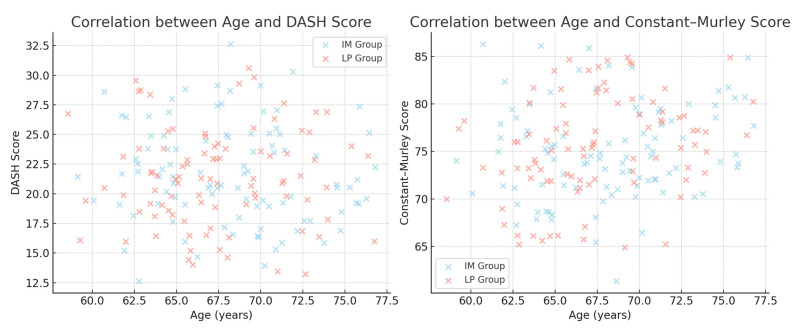
Scatter plots showing the correlation between patient age and functional scores. (**Left**) No significant correlation between age and DASH score (*p* = 0.986). (**Right**) No significant correlation between age and Constant–Murley Score (*p* = 0.565).

The correlations are shown in Figure 4.

### 3.5. Range of Motion (ROM) Recovery

The distribution of these scores is illustrated in Figure 2 and Figure 3, where the boxplots illustrate the distribution of DASH and Constant–Murley scores across the two groups. The recovery of shoulder ROM was evaluated at 1, 3, 6, and 12 months postoperatively.

- Flexion at 12 months: The IM group had a mean of 132.5° ± 8.1°, while the LP group had 131.8° ± 7.9° (*p* = 0.940).
- Abduction at 12 months: The IM group had a mean of 122.3° ± 9.3°, while the LP group had 121.6° ± 8.7° (*p* = 0.888).
- Internal Rotation at 12 months: The IM group had a mean of 96.2° ± 10.2°, while the LP group had 94.0° ± 9.8° (*p* = 0.061).
- External Rotation at 12 months: The IM group had a mean of 105.8° ± 9.1°, while the LP group had 103.5° ± 8.6° (*p* = 0.150).

**Figure 5:**
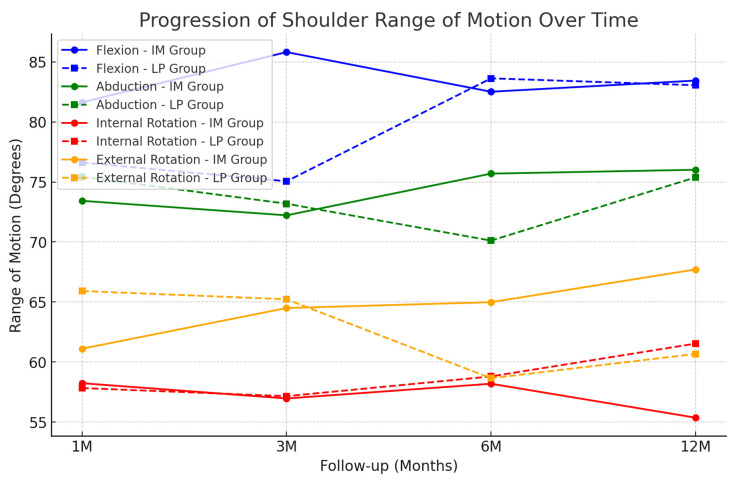
Line graph illustrating the progression of shoulder range of motion (flexion, abduction, internal rotation, and external rotation) over 1, 3, 6, and 12 months postoperatively. The recovery trend was similar in both groups for all movements.

No statistically significant differences were observed between the two groups in terms of shoulder ROM at any time point. The progression of shoulder motion is shown in Figure 5.

To further evaluate the impact of fracture severity on postoperative shoulder mobility, ROM values were stratified according to the Neer classification. As shown in Table 5, patients with two-part fractures achieved higher ROM values compared to those with three- and four-part fractures. However, within each fracture category, no statistically significant differences were found between treatment groups. For instance, in two-part fractures, forward flexion averaged 138.2 ± 6.5° (IM) vs. 136.4 ± 6.8° (LP) (*p* = 0.352), and abduction averaged 129.0 ± 7.2° (IM) vs. 127.2 ± 7.5° (LP) (*p* = 0.401).

These findings suggest that both surgical techniques allow the satisfactory recovery of shoulder mobility, and that fracture complexity rather than fixation method may play a more relevant role in the final ROM outcome.

### 3.6. Complications

A total of 22 patients (11.8%) experienced at least one postoperative complication, with no statistically significant difference between the intramedullary nail (IM) and locking plate (LP) groups (10.4% vs. 13.2%, *p* = 0.625), as detailed in Table 6.

The most frequent complications were hardware-related issues (4.2% in IM vs. 5.5% in LP), followed by malunion (3.1% vs. 3.3%) and infections (2.1% vs. 3.3%). Non-unions occurred in one patient in the IM group and two patients in the LP group. Avascular necrosis (AVN) of the humeral head was observed in two cases treated with IM and three with LP, all of which occurred in four-part fractures.

Six patients required a secondary surgical procedure: three in the IM group and four in the LP group. Indications for reoperation included hardware removal, deep infection, and revision for AVN. No implant-related catastrophic failures or neurovascular complications were reported in either group.

When stratifying complications by fracture type, avascular necrosis (AVN) was exclusively observed in patients with four-part fractures. Specifically, AVN occurred in 2 of 21 patients (9.5%) in the IM group and 3 of 26 patients (11.5%) in the LP group. No cases of AVN were reported in two- or three-part fractures (Table 7).

## 4. Discussion

Proximal humerus fractures (PHFs) are frequent in elderly populations and continue to represent a therapeutic challenge, particularly in the setting of osteoporotic bone. Among surgical options, intramedullary nailing (IM) and locking plate (LP) fixation are the most widely adopted techniques; however, consensus regarding their respective indications remains lacking [7,8].

### 4.1. Comparison with Existing Literature

Our study demonstrates that both IM and LP fixation result in comparable functional outcomes at 12 months, as assessed by the DASH (*p* = 0.484) and Constant–Murley (*p* = 0.057) scores. These findings are consistent with prior literature. In particular, Sun et al. reported no significant differences in functional outcomes between these two techniques in a meta-analysis of 13 studies [15]. Similarly, Gracitelli et al. and Maier et al. found no clinically relevant superiority of one technique over the other [11,16].

In contrast, some studies suggest that LP fixation may offer superior early functional outcomes. Zhu et al. observed significantly higher ASES scores and supraspinatus strength in the LP group at one year of follow-up, although these differences disappeared at three years [17]. This suggests that, while LP fixation might provide an initial advantage, long-term outcomes tend to converge.

Regarding the range of motion (ROM) recovery, our data show no significant differences between IM and LP groups at 1, 3, 6, and 12 months. Hettrich et al. and Maier et al. similarly found that both fixation methods allow for comparable ROM restoration at one year [11,18]. These findings reinforce the idea that both techniques enable satisfactory functional recovery, with no clear superiority of one over the other.

Importantly, unlike most previous studies, we performed a stratified analysis by fracture complexity according to the Neer classification. In two-part fractures, both techniques yielded excellent and equivalent outcomes. In three- and four-part fractures, a slight trend toward improved Constant scores was noted in the LP group, though not statistically significant. This stratification adds clinical nuance and strengthens the interpretation of our findings.

### 4.2. Age, Bone Quality, and Confounding Factors

An age difference between the two groups was initially observed (IM: 68.2 vs. LP: 62.4 years, *p* < 0.001); however, age was not correlated with functional recovery (DASH: r = 0.001, *p* = 0.986; CMS: r = −0.042, *p* = 0.565). We extended our analysis beyond age and included bone quality (via the Deltoid Tuberosity Index) and smoking status as confounding variables in multivariable models. None significantly affected functional outcomes. This supports the hypothesis that patient-specific factors, rather than age alone, should guide treatment selection [9,19].

### 4.3. Complications and Radiographic Outcomes

We reported a complication rate of 11.8% with no statistically significant difference between the IM and LP groups. The most frequent complications were hardware-related (e.g., screw loosening, implant migration), followed by non-union and avascular necrosis (AVN), which occurred primarily in four-part fractures. Revision surgery was required in a small subset of cases without group differences. These results highlight the importance of fracture severity—rather than implant type—as a predictor of adverse outcomes [3].

Healing was achieved in the vast majority of cases, with comparable union rates between groups. No significant difference was observed in the incidence of malunion or AVN, although these outcomes remained more common in complex fracture patterns. Our subgroup analysis confirms that AVN occurred exclusively in four-part fractures, with similar incidence between the IM and LP groups. These findings suggest that the risk of AVN is more closely related to fracture complexity than to the choice of fixation method.

### 4.4. Surgical Indications and Biomechanical Considerations

Our study reflects real-world decision-making: implant selection was based on fracture morphology, bone quality, and surgeon experience, with both techniques being used across all fracture types. Biomechanical studies have highlighted key theoretical differences between intramedullary nailing (IM) and locking plate (LP) fixation.

Locking plates provide rigid fixation and allow for anatomical reduction, making them the preferred option for comminuted fractures and fractures with medial cortical loss [20]. However, this method requires extensive soft tissue dissection, which may compromise vascular supply and has been associated with an increased risk of avascular necrosis [21]. In addition, recent evidence has shown that plate osteosynthesis can trigger a more pronounced systemic inflammatory response compared to intramedullary nailing. Moldovan (2024) reported significantly higher serum levels of inflammatory cytokines, such as IL-6 and TNF-α, following locking plate fixation for humeral shaft fractures [22]. This enhanced inflammatory reaction may contribute to increased postoperative pain, swelling, and delayed rehabilitation, particularly in elderly patients with reduced physiological reserve.

Intramedullary nails, on the other hand, offer a less invasive approach that preserves periosteal blood supply and reduces soft tissue trauma [23]. Despite these advantages, potential drawbacks include rotator cuff irritation and subacromial impingement, which may negatively impact postoperative shoulder function [24].

Despite the biomechanical and theoretical differences between the two techniques, our clinical findings do not support a clear superiority of one implant over the other within the first year of follow-up.

### 4.5. Conservative Treatment and External Validity

We acknowledge the growing interest in non-operative treatments for displaced PHFs in elderly patients. However, all patients in our cohort had fractures deemed unsuitable for conservative management, as defined by significant displacement (Neer ≥ 2-part) and clinical decision-making at presentation. Although our study does not include a non-operative control group, future research should consider including such arms for comprehensive comparison.

### 4.6. Study Limitations and Future Directions

This study has several limitations. The retrospective design introduces potential selection bias, and treatment allocation was not randomized. While we addressed key confounding variables statistically, unmeasured confounders (e.g., surgeon experience, compliance with rehabilitation) may persist. The follow-up duration of 12 months may not capture late complications such as implant loosening, arthritis, or delayed AVN.

Future prospective studies should account for the following:

- Include systematic bone mineral density (BMD) assessments;
- Compare outcomes with conservative treatment in selected patients;
- Extend follow-up beyond 12 months;
- Consider matching or stratification for fracture pattern and surgeon variables.

## 5. Conclusions

In this cohort of elderly patients with displaced proximal humerus fractures, both intramedullary nails and locking plates provided comparable results in terms of functional recovery, ROM, and complication rates. Age and fracture complexity did not appear to significantly influence clinical outcomes when accounting for confounding variables. Given these findings, the choice of fixation method should be individualized based on fracture morphology, bone quality, and surgical expertise rather than predefined protocols.

## Figures and Tables

**Table 1:** Demographic and clinical characteristics of the study population.

Variable	IM Group (*n* = 96)	LP Group (*n* = 91)	*p*-Value
Sample size	96	91	–
Mean age (years)	68.2 ± 7.0	62.4 ± 9.0	<0.001
Gender distribution (male/female)	39/57	35/56	0.879
Neer two-part fractures	35 (36.5%)	25 (27.5%)	0.190
Neer three-part fractures	40 (41.5%)	40 (44.0%)	0.856
Neer four-part fractures	21 (22.0%)	26 (28.5%)	0.514
Right side involvement (%)	51 (53.1%)	47 (51.6%)	0.831
Dominant side involvement (%)	39 (40.6%)	38 (41.8%)	0.872
Low-energy trauma (%)	82 (85.4%)	75 (82.4%)	0.579
Diabetes mellitus (%)	14 (14.6%)	12 (13.2%)	0.798
Osteoporosis treatment (%)	18 (18.8%)	14 (15.4%)	0.561
DTI < 1.4 (%) (low bone density)	32 (33.3%)	21 (23.1%)	0.108
ASA III–IV (%)	40 (41.7%)	28 (30.8%)	0.131
Mean BMI (kg/m^2^)	26.8 ± 3.4	27.1 ± 3.2	0.541
Mean time to surgery (days)	5.1 ± 1.3	5.3 ± 1.4	0.392

Note. IM = Intramedullary Nail; LP = Locking Plate; ASA = American Society of Anesthesiologists; DTI = Deltoid Tuberosity Index; BMI = Body Mass Index; M/F = Male/Female; SD = Standard Deviation.

**Table 2:** Overall functional outcomes.

Score	Group	Mean ± SD	95% CI	*p*-Value
DASH Score	IM	20.6 ± 6.1	(19.4, 21.8)	0.484
	LP	21.2 ± 5.3	(20.1, 22.3)	
Constant–Murley Score	IM	79.6 ± 8.0	(78.0, 81.3)	0.057
	LP	77.5 ± 7.6	(75.9, 79.0)	

Note. IM = Intramedullary Nail; LP = Locking Plate; DASH = Disabilities of the Arm, Shoulder, and Hand; CMS = Constant–Murley Score; SD = Standard Deviation; CI = Confidence Interval.

**Table 3:** Functional outcomes stratified by Neer classification.

Neer Type	Score	IM Group (*n*)	LP Group (*n*)	*p*-Value
two-part	DASH	19.8 ± 5.2 (*n* = 35)	20.3 ± 5.5 (*n* = 25)	0.642
	CMS	82.1 ± 6.2	80.5 ± 6.8	0.318
three-part	DASH	21.3 ± 5.7 (*n* = 40)	21.0 ± 5.4 (*n* = 40)	0.785
	CMS	78.3 ± 7.1	77.9 ± 7.5	0.812
four-part	DASH	22.4 ± 6.1 (*n* = 21)	23.1 ± 6.5 (*n* = 26)	0.571
	CMS	76.0 ± 7.9	74.5 ± 8.2	0.402

Note. IM = Intramedullary Nail; LP = Locking Plate; DASH = Disabilities of the Arm, Shoulder, and Hand; CMS = Constant–Murley Score; SD = Standard Deviation.

**Table 4:** Functional outcomes stratified by age group.

Age Group	Score	IM Group	LP Group	*p*-Value
<65 years	DASH	20.1 ± 5.2	20.6 ± 5.5	0.641
	CMS	80.8 ± 6.9	80.2 ± 7.1	0.578
≥65 years	DASH	21.9 ± 5.8	22.0 ± 5.9	0.934
	CMS	77.5 ± 8.1	76.2 ± 8.7	0.401

Note. IM = Intramedullary Nail; LP = Locking Plate; DASH = Disabilities of the Arm, Shoulder, and Hand; CMS = Constant–Murley Score; SD = Standard Deviation.

**Table 5:** Range of motion at 12 months stratified by Neer classification.

Neer Type	ROM	IM Group (Mean ± SD)	LP Group (Mean ± SD)	*p*-Value
Two-part	Flexion (°)	138.2 ± 6.5	136.4 ± 6.8	0.352
	Abduction (°)	129.0 ± 7.2	127.2 ± 7.5	0.401
	External Rotation (°)	51.6 ± 5.8	50.8 ± 5.9	0.612
	Internal Rotation (°)	56.2 ± 6.1	55.3 ± 6.3	0.537
Three-part	Flexion (°)	132.0 ± 8.4	131.2 ± 8.2	0.692
	Abduction (°)	121.5 ± 9.1	120.8 ± 9.0	0.773
	External Rotation (°)	47.4 ± 6.2	46.9 ± 6.3	0.823
	Internal Rotation (°)	53.1 ± 6.7	52.4 ± 6.6	0.742
Four-part	Flexion (°)	125.6 ± 7.8	124.1 ± 7.9	0.586
	Abduction (°)	116.4 ± 8.5	115.7 ± 8.6	0.723
	External Rotation (°)	43.9 ± 6.0	43.1 ± 6.2	0.690
	Internal Rotation (°)	49.7 ± 6.3	48.9 ± 6.4	0.603

Note. IM = Intramedullary Nail; LP = Locking Plate; ROM = Range of Motion; SD = Standard Deviation; ° = Degrees.

**Table 6:** Postoperative complications stratified by treatment group.

Complication	IM Group (*n* = 96)	LP Group (*n* = 91)	*p*-Value
Infection (superficial/deep)	2 (2.1%)	3 (3.3%)	0.675
Non-union	1 (1.0%)	2 (2.2%)	0.552
Malunion	3 (3.1%)	3 (3.3%)	0.946
Avascular necrosis (AVN)	2 (2.1%)	3 (3.3%)	0.675
Hardware failure (e.g., screw/backout, nail migration)	4 (4.2%)	5 (5.5%)	0.726
Reoperation (any cause)	3 (3.1%)	4 (4.4%)	0.697
Total patients with ≥1 complication	10 (10.4%)	12 (13.2%)	0.625

**Note.** IM = Intramedullary Nail; LP = Locking Plate; AVN = Avascular Necrosis; *n* = Number of patients.

**Table 7:** Incidence of AVN in four-part fractures by treatment group.

Group	No. of Four-Part Fractures	AVN Cases	Incidence (%)
IM Group	21	2	9.5%
LP Group	26	3	11.5%

**Note.** AVN = Avascular Necrosis; IM = Intramedullary Nail; LP = Locking Plate.

## Data Availability

Photos of the surgical techniques are available upon request.

## Abbreviations

The following abbreviations are used in this manuscript:

PHFs	Proximal humerus fractures
IM	Intramedullary nails
LP	Locking plates
BMD	Bone mineral density
ROM	Range of motion
DASH	Disabilities of the Arm, Shoulder and Hand
CMS	Constant–Murley Score
